# Regional machine learning-based estimation of methane emissions from rice cultivation in South Korea

**DOI:** 10.1038/s41598-026-49883-4

**Published:** 2026-04-27

**Authors:** Hyoungseok Lee, Jongmun Lee, Sora Lee, Hyeran Park, Minji Lee, Youngjae Jeong

**Affiliations:** Rural Development Administration, National Institute of Agricultural Sciences, Wanju, 55365 Republic of Korea

**Keywords:** Methane emissions, Rice paddies, Machine learning, Greenhouse gas inventory, Regional modeling, Climate sciences, Environmental sciences

## Abstract

Machine learning approaches, XGBoost (XGB) and Random Forest (RF), were applied to estimate methane (CH₄) emissions from paddy fields in Gimje, South Korea, using three years of chamber-based observations. Both models achieved Nash–Sutcliffe efficiency (NSE) values above 0.5, indicating acceptatble predictive performance. XGB better captured extreme values and temporal variability, whereas RF reproduced mean emission trends more reliably, highlighting complementary strengths. Regional simulations produced mean emission factors of 2.64 and 2.35 kg CH₄ ha⁻1 d⁻1 for XGB and RF, respectively, comparable to or slightly higher than the Tier 2 country-specific factor (2.32 kg CH₄ ha⁻1 d⁻1) currently used in Korea’s national greenhouse gas inventory. Spatially, XGB identified high-emission hotspots with greater sensitivity, while RF yielded smoother patterns with lower uncertainty. Model-based regional estimates (6.68–7.51 Gg CH₄ yr⁻1) showed uncertainty ranges of ± 12–13%, substantially lower than the ± 50% default suggested by IPCC guidelines, underscoring the models’ robustness. These results demonstrate the feasibility of applying machine learning to CH₄ estimation at regional scales, thereby addressing the limitations of uniform Tier 2 emission factors. Incorporating region-specific, data-driven approaches can improve inventory accuracy and provide a stronger scientific basis for mitigation strategies. This study highlights the potential of machine learning models to complement existing methods and contribute to the advancement toward a Tier 3 inventory framework for agricultural greenhouse gas reporting.

**Supplementary Information:**

The online version contains supplementary material available at 10.1038/s41598-026-49883-4.

## Introduction

Methane (CH₄) is a major greenhouse gas with a global warming potential (GWP) that is approximately 28 times higher than that of carbon dioxide (CO₂) over a 100-year time horizon when accounting for atmospheric absorption and radiative forcing^1^. Although its atmospheric lifetime is relatively short, its strong radiative forcing makes CH₄ a significant contributor to global warming^2^. In 2017, agricultural CH₄ emissions were estimated at 198–232 Tg CH₄ yr⁻1, accounting for 22–39% of global emissions^3^. Among these, rice cultivation contributes approximately 24–40 Tg CH₄ yr⁻1^3,4^. Methane emissions from paddy fields occur primarily under anaerobic conditions and vary spatially and temporally^5^. Emissions are concentrated at specific growth stages, such as tillering and heading^6–8^, and are influenced by interactions among water management, organic matter application, soil conditions, and cultivar characteristics^9,10^ (Yagi and Minami 1990a).

Since 2011, Korea has developed a national greenhouse gas inventory and, since 2023, has fully adopted the 2006 IPCC Guidelines for emission estimation^11^. According to the IPCC guidelines, country-specific emission factors are classified as Tier 2, whereas advanced methods based on long-term observations or modelling are considered Tier 3^12^. By 2022, Korea’s total CH₄ emissions were reported as 35,150 Gg CO_2_-eq. yr⁻1, of which agriculture accounts for 17,365 Gg CO_2_-eq. yr⁻1, which is nearly half the total. Rice paddies alone emitted 7,112 Gg CO_2_-eq. yr⁻1, representing 30.1% of agricultural CH₄ emissions and 20.2% of the national total^11^. Thus, rice cultivation in Korea is a major source of methane emissions and improving the accuracy of emission estimates is critical for developing effective mitigation strategies. However, the current Tier 2 approach applies a default emission factor developed from short-term experiments with limited regions and cultivars uniformly across the country, which inherently fails to reflect regional diversity and actual cultivation practices.

Process-based models, such as DNDC and CH4MOD, have been applied as Tier 3 approaches for agricultural GHG estimation^13–15^. In Korea, models such as DNDC and DayCent have been tested against field observations, but their applicability is limited owing to inconsistencies with local agricultural environments, highlighting the need for model structure improvement and extensive calibration data^16^. More recently, studies in East Asia have reported the application of machine learning models for spatially explicit estimation of CH₄ emissions from rice paddies. These models have demonstrated advantages in capturing complex interactions among multiple factors that influence emissions, thereby improving prediction accuracy^17–20^. Although machine learning models are less interpretable than process-based models, they effectively capture the nonlinear relationships among diverse input variables^21,22^. XGBoost (extreme gradient boosting,XGB) In particular, XGB has shown superior performance in estimating global CH₄ emissions due to its high accuracy and ability to handle missing values, outperforming models such as support vector regression (SVR), LightGBM, and random forest (RF)^23^. RF, on the other hand, is less prone to overfitting and robust in estimating mean values and has been widely applied to CH₄ emission estimation from rice paddies and remote sensing–based studies^19,21,24^.

This study aimed to evaluate the applicability of machine learning models for spatially explicit estimation of CH₄ emissions from rice paddies and to assess regional consistency with Korea’s Tier 2 emission factors. Using chamber-based field measurements collected in Gimje from 2022 to 2024, we developed and validated the XGB and RF models, applied them to the regional scale, and estimated the annual emissions and emission factors. Furthermore, the derived emission factors were compared with the national Tier 2 default factor to evaluate the validity of the machine learning-based regional approach and its potential contribution to improving the national inventory. This study suggests that field-based machine learning models can improve regional estimation consistency and provide methodological insight toward Tier 3 level inventory development.

## Materials and methods

### Study area

**Fig. 1:**
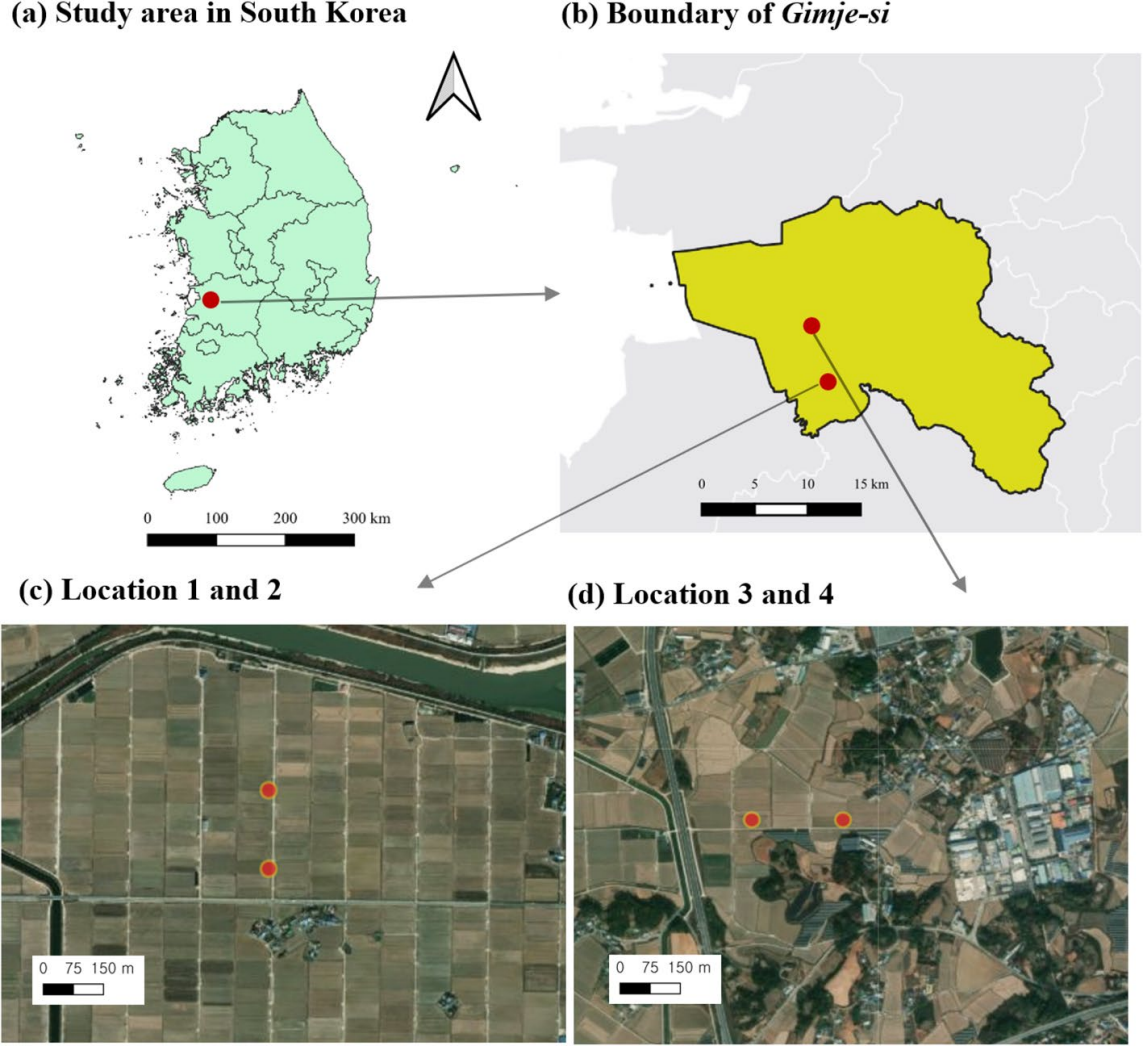
(**a**) Location of the study area in South Korea. (**b**) Administrative boundary of Gimje-si. (**c**–**d**) High-resolution satellite imagery showing clustered sampling locations in Buryang-myeon (L1 and L2) and Myeongdeok-dong (L3 and L4). Maps were created using R (version 4.3.1) with the leaflet package (version 2.2.2; https://rstudio.github.io/leaflet/). Satellite basemap: Esri World Imagery © Esri, Maxar, Earthstar Geographics, and the GIS User Community (https://www.arcgis.com/home/item.html?id=10df2279f9684e4a9f6a7f08febac2a9).

Gimje-si, Jeollabuk-do, has 20,815 ha of paddy fields according to the 2022 Agricultural Area Survey conducted by the Korean Statistical Information Service^25^*,* making it the second largest area within the province (see Fig. 1). Field observations were conducted from 2022 to 2024 in Buryang-myeon (L1 and L2) and Myeongdeok-dong (L3 and L4), as summarized in Table 1. These sites represent typical rice-growing areas that have been continuously cultivated for more than 20 years. The rice cultivars used in the experiments were Chamdongjin, Shindongjin, and Dongjinchal, the major varieties traditionally cultivated by local farmers (see Table 1).

**Table 1:** Description of observation sites.

Year	Location	Soil texture	Planting date	Rice cultivar	Water management	Cases
2022	L1	Sandy loam	05/19	Dongjinchal (Oryza sativa L.)	CF, MD, MD + AWD	9
2022	L2	Sandy clay loam	05/19	Dongjinchal (Oryza sativa L.)	MD, MD + AWD	9
2023	L1	Sandy loam	05/25	Chamdongjin (Oryza sativa L.)	CF, MD, MD + AWD	9
2023	L2	Sandy clay loam	05/25	Chamdongjin (Oryza sativa L.)	MD, MD + AWD	6
2024	L3	Sandy clay loam	05/20	Shindongjin (Oryza sativa L.)	CF, MD, MD + AWD	15
2024	L4	Sandy clay loam	05/20	Shindongjin (Oryza sativa L.)	CF	12

L1 (35.755°N, 126.869°E); L2 (35.753°N, 126.869°E); L3 (35.798°N, 126.847°E); L4 (35.798°N, 126.844°E); Cases denote the number of sampling units (experimental plots) included in the analysis for each year-location combination; CF, continuous flooding (fields remained flooded throughout the growing season); MD, mid-drainage (approximately 30 days of drainage after transplanting); MD + AWD, mid-drainage followed by alternate wetting and drying after the heading stage; Standard cultivation practices were consistently applied across all plots: chemical nitrogen (N) fertilizer was applied at 130 kg N ha⁻^1^, and rice straw was completely removed after harvest to minimize external organic matter inputs; In 2024 (L4), field was converted from soybean cultivation (paddy–upland rotation). Mixed livestock manure compost was applied, and the chemical N fertilizer rate was reduced accordingly

### Methane sampling and analysis

Methane (CH₄) emissions were measured using the closed-chamber method, which is consistent with the approach applied to the development of national emission factors in Korea^26,27^. The chamber was a transparent polycarbonate rectangular box of dimensions 60 × 60 × 130 cm. It was equipped with top and side openings, a ventilation fan, a power supply unit, a thermometer holder, a sampling port, and a drainage outlet at the base^26^. The chamber was installed by inserting four supporting legs directly into the soil, ensuring stable placement and a tight seal at the soil–chamber interface without the use of separate soil collars. Gas samples were collected using a 60 mL syringe, and an initial sample was taken at 0 min, after which the chamber was sealed. Prior to sealing, the drainage outlet at the base was closed with a rubber stopper to prevent gas leakage during the enclosure period. Following a 30 min enclosure, the fan was operated for 1 min to ensure air circulation, and the final sample was collected. During sampling, the chamber air temperature and headspace height were recorded. During gas sampling, the chamber was fully closed (top and side panels sealed) to ensure a closed system. Outside the sampling period, the top and side panels were opened to minimize potential interference with rice growth. Collected gas samples were analyzed using gas chromatography (Agilent 7890B), and CH₄ concentrations were quantified with a flame ionization detector (FID). The measured concentration changes were converted to fluxes per unit area and time, and cumulative emissions were calculated using the following equation^28–31^.

1 $$F=\left(\rho \times V/A\right)\times \left(\Delta c/\Delta t\right)\times \left(273/T\right)$$

where *F* is the CH₄ flux (mg m⁻2 h⁻1), *ρ* is the pressure (g cm⁻3), *V* is the chamber volume (m3), *A* is the chamber surface area (m2), *Δc/Δt* is the rate of increase in CH₄ concentration inside the chamber (mg CH₄ m⁻3 h⁻1), and *T* is the absolute temperature (K), calculated as 273 plus the mean chamber temperature (°C).

2 $$\text{Total C}{\mathrm{H}}_{4}\text{ flux}={\sum }_{\mathrm{i}=1}^{\mathrm{n}}\left({\mathrm{F}}_{\mathrm{i}}\times {\mathrm{D}}_{\mathrm{i}}\right)$$

where *F*_i_ is the rate of flux (g m⁻2 d⁻1) in the *i*^*th*^ sampling interval, *D*_i_ is the number of days in the *i*^*th*^ sampling interval, and *n* is the total number of sampling intervals.

Supplementary Table S1 describes the water management regimes applied at each site. An overview of observed CH₄ flux dynamics is provided in Supplementary Fig. S1, with seasonal mean (± SD) values summarized in Supplementary Table S2. These results describe the magnitude and variability of methane emissions across site–year combinations and water management regimes prior to model evaluation.

### Model description

The XGBoost (XGB) and Random Forest (RF) models were trained using the CH₄ flux field observation dataset collected at the four study sites (L1–L4) from 2022 to 2024. Field-measured CH₄ fluxes were used as the dependent variables, whereas meteorological, soil, crop growth, management, remote sensing indices, and topographic factors were used as independent variables. Meteorological variables included daily precipitation and mean air temperature obtained from the Korea Meteorological Administration^32^. Soil variables were represented by soil texture (sand, silt, and clay) measured at the experimental sites. Crop growth was represented as days after transplanting (DAT), with a standard cultivation period of 137 days applied for harvest, which is consistent with the national inventory^11^. Grain yield (gy) was determined by air-drying harvested rice straw in the shade and converting it to a 15% moisture content. Management factors reflect water management practices, classified as flooding or drainage. Remote sensing indices included MODIS EVI from GEE^33^ and ERA5-Land climate data^34^. In addition, a digital elevation model (DEM, 90 m) was used as a topographic variable^35^.

### Model evaluation

Model performance was evaluated using the coefficient of determination (R2), root mean square error (RMSE), Nash–Sutcliffe efficiency (NSE), and percent bias (PBIAS)^36^. R2 indicates the explanatory power of the model, RMSE reflects the magnitude of the prediction error, NSE represents the efficiency of reproducing the temporal variability, and PBIAS indicates whether the model tends to overestimate or underestimate. For model validation, the dataset was randomly split into 80% for calibration and 20% for validation, and this procedure was repeated ten times to reduce potential bias from a single split.

3 $${R}^{2}={\left(cor\left(pred,obs\right)\right)}^{2}$$

4 $$RMSE=\sqrt{\frac{1}{n}{\sum }_{i=1}^{n}{\left(ob{s}_{i}-pre{d}_{i}\right)}^{2}}$$

5 $$NSE=1-\frac{{\sum }_{i=1}^{n}{\left(ob{s}_{i}-pre{d}_{i}\right)}^{2}}{{\sum }_{i=1}^{n}{\left(ob{s}_{i}-\overline{obs }\right)}^{2}}$$

6 $$PBIAS\left(\mathrm{\%}\right)=100\times \frac{{\sum }_{i=1}^{n}\left(ob{s}_{i}-pre{d}_{i}\right)}{{\sum }_{i=1}^{n}ob{s}_{i}}$$

where obs_i_ and pred_i_ are the observed and predicted CH₄ fluxes at site *i*, respectively, and *n* is the total number of samples.

To prevent overfitting and improve generalization performance, hyperparameter optimization was performed using a grid search for both models. For XGB, parameters such as *max_depth*, *eta*, *subsample*, *colsample_bytree*, *min_child_weight*, *lambda*, and *alpha* were tuned, whereas for RF, the number of trees (*ntree*) and sampled variables (*mtry*) were optimized. Cross-validation was applied in parallel and the parameters were selected to minimize discrepancies between the calibration and validation metrics. An ensemble approach was applied to quantify the uncertainty of XGB- and RF-based predictions. Multiple models were trained by growth stage (rooting, tillering, reproductive, and ripening) using repeated datasets and predictions were generated for the same input variables. The ensemble mean was presented as the final prediction, whereas the uncertainty was expressed as the standard deviation of the repeated predictions. A 95% confidence interval was calculated as the mean ± 1.96 × standard deviation, providing both lower and upper bounds for each pixel-level prediction. This approach allowed us to incorporate variability arising from data partitioning and to explicitly represent the uncertainty of regional CH₄ emission estimates.

To identify the dominant predictors of CH₄ emissions, variable importance was extracted from the trained machine learning models. For the RF model, importance was quantified using the percentage increase in mean squared error (%IncMSE), based on permutation importance, which measures the reduction in model accuracy when a predictor is randomly permuted^21,37^. For XGB model, importance was evaluated using the Gain metric, representing the relative contribution of each feature to the reduction of the loss function across all tree splits^38^. Importance values were calculated for each of the 10 repeated 8:2 train–validation splits and then averaged to reduce variability associated with random data partitioning.

### Regional prediction

Regional CH₄ emission prediction was conducted using the trained stage-specific RF and XGB models. The spatial preprocessing and per-pixel prediction workflow were implemented in R, and the scripts are publicly available at GitHub (https://github.com/lhs0218/gimje_ch4). For the prediction stage, the same predictor framework described in Table 2 was applied but extended to the regional-scale CH₄ emission estimation for Gimje. Meteorological and remote sensing data were obtained from the same sources as in the training phase, whereas soil information was derived from the Soil Information System (1:25,000 detailed soil map) and national soil database. Grain yield data were obtained from the 2022 Crop Production Survey provided by the Korean Statistical Information Service^25^. A representative regional yield value was uniformly applied to all paddy pixels. Water management was parameterized as continuous flooding (CF) using a predefined DAT-based seasonal schedule, and the same management regime was uniformly applied across the study area. All spatial data were processed using QGIS v3.34.3 and R v4.3.1 (terra/sf packages), standardized to EPSG:5179, and resampled to a resolution of 270 m. Paddy areas were masked using the 2022 Farm Map^39^, and CH₄ emissions were predicted on a per-pixel and daily basis. Total CH₄ emissions for Gimje were estimated by aggregating daily predictions over the cultivation period and summing emissions across the effective paddy area (20,769 ha) defined by the Farm Map during the cultivation period, from which area-based emission factors (g m⁻2 day⁻1) were also derived (as summarized in Table 2).

**Table 2:** Summary of predictor variables used in the models.

Variables	Unit	Source (Training)	Source (Prediction)	Description
DAT	days	Field survey	Calculated from transplanting date	Days after transplanting
tavg	℃	KMA	KMA (daily raster data)	Daily mean air temperature
rain_mm	mm	KMA	KMA (daily raster data)	Daily precipitation
EVI	-	MODIS (GEE)	MODIS (GEE)	Enhanced vegetation Index
surface_net_solar_radiation	MJ m^-2^	ERA5-Land (GEE)	ERA5-Land (GEE)	Net surface solar radiation
volumetric_soil_water	m^3^ m^-3^	ERA5-Land (GEE)	ERA5-Land (GEE)	Soil moisture (top layer)
water_management	-	Field survey	Predefined CF schedule	Flooding/drainage type
sand	%	Field survey	Soil Information System (1:25,000)	Sand fraction
silt	%	Field survey	Soil Information System (1:25,000)	Silt fraction
clay	%	Field survey	Soil Information System (1:25,000)	Clay fraction
gy	kg ha^-1^	Field survey	KOSIS 2022 (regional mean)	Yield indicator
elevation	m	DEM (90 m)	DEM (resampled raster)	Topographic elevation

In this study, growth stages were assumed based on days after transplanting (DAT): Rooting (≤ 10 DAT), Tillering (10 < DAT ≤ 45), Reproductive (45 < DAT ≤ 90), and Ripening (90 < DAT ≤ 137).

## Results and discussion

### Model training and validation with field observations

The results summarized in Table 3 present the validation performance across growth stages (rooting, tillering, reproductive, and ripening) and their overall metrics. Both XGB (XGBoost) and RF (Random Forest) achieved Nash–Sutcliffe efficiency (NSE) values above 0.5, indicating an acceptable level of predictive performance based on the NSE classification criteria^40,41^. A closer examination of the metrics revealed the differences between the two models. During validation, XGB exhibited superior performance, with a lower RMSE (0.115 g m⁻^2^ d⁻^1^), higher R^2^ (0.734), and higher NSE (0.725), demonstrating better predictive accuracy and the ability to reproduce temporal variability than RF. These results are consistent with previous findings that XGB effectively captures complex drivers in paddy carbon models and delivers high predictive performance under diverse conditions^17,42^, underscoring its strength in modelling interactions in agricultural ecosystems. However, XGB showed a relatively high PBIAS (2.53%), suggesting potential over- or underestimation under certain conditions. In contrast, RF showed a lower overall performance (RMSE = 0.134, R2 = 0.666, NSE = 0.664) than XGB, but its PBIAS was extremely small (0.095), indicating a minimal deviation between the predicted and observed means. This reflects the RF’s strength in capturing the overall average trends, which can be attributed to its bagging-based learning structure and independence among individual trees^21^. Furthermore, studies in climate modelling have reported cases where RF outperformed XGB or LightGBM^18^, suggesting that RF may serve as a competitive alternative, depending on the limited training data and highly variable datasets.

**Table 3:** Summary of predictor variables used in the models.

Stage	Model	RMSE	R^2^	NSE	PBIAS
Rooting	XGB	0.035	0.226	-1.846	146.181
Rooting	RF	0.047	0.128	-3.535	123.94
Tillering	XGB	0.139	0.476	0.333	6.574
Tillering	RF	0.141	0.475	0.433	0.875
Reproductive	XGB	0.115	0.77	0.765	-0.582
Reproductive	RF	0.139	0.674	0.666	-0.392
Ripening	XGB	0.131	0.676	0.656	1.881
Ripening	RF	0.13	0.677	0.663	1.2
Overall	XGB	0.115	0.734	0.725	2.527
Overall	RF	0.134	0.666	0.664	0.095

RMSE = root mean square error (g m⁻^2^ d⁻^1^); R^2^ = coefficient of determination; NSE = Nash–Sutcliffe efficiency; PBIAS = percentage bias.

**Fig. 2:**
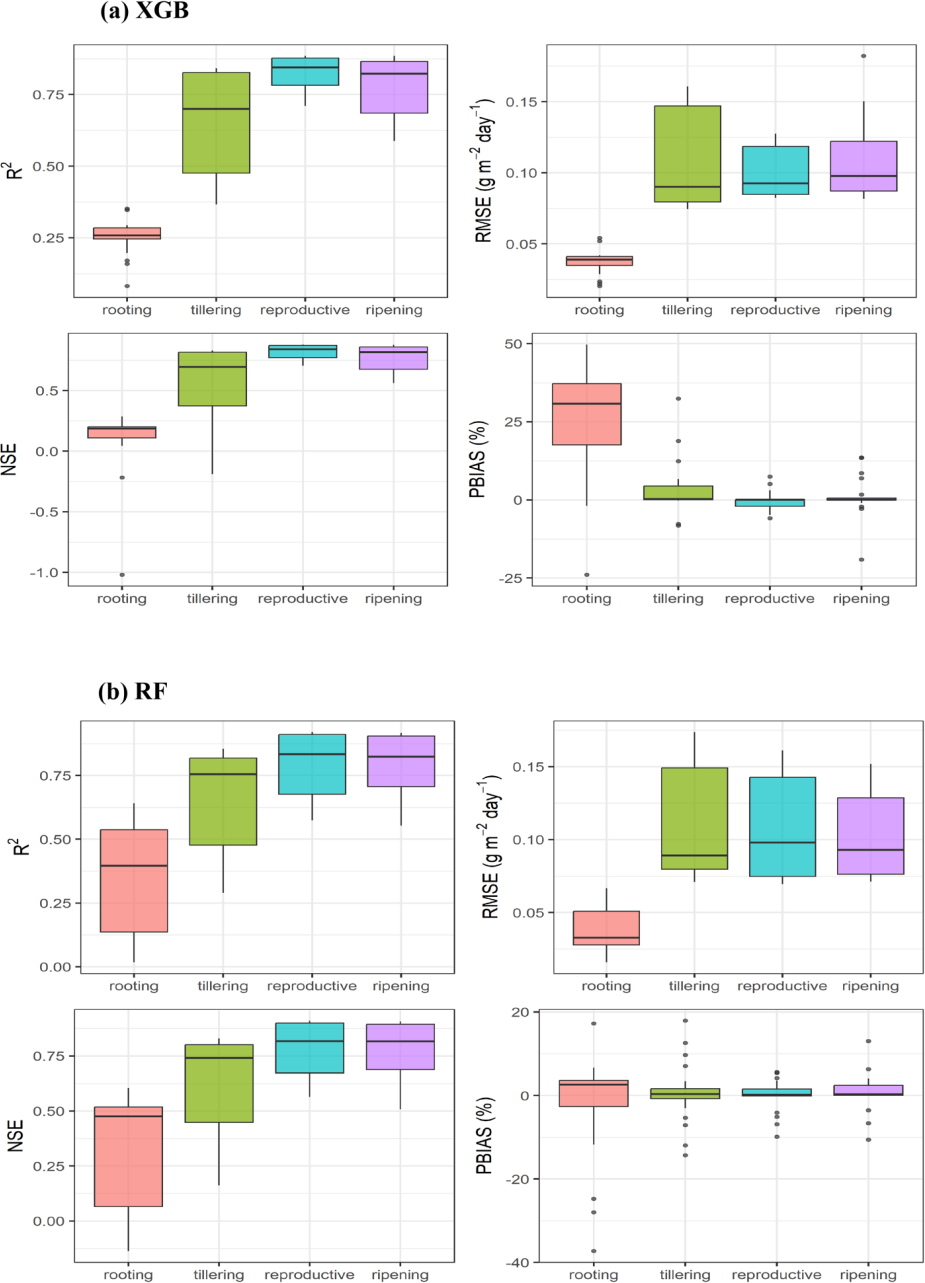
Evaluation of (**a**) XGB and (**b**) RF model accuracy at different rice growth stages, based on R2, RMSE, NSE, and PBIAS. Each box plot represents the distribution of performance metrics derived from ten repeated 8:2 random splits of the validation dataset.

The distribution of performance metrics by growth stage (as illustrated in Fig. 2), indicated that both models achieved stable predictive power during the reproductive and ripening stages. The relatively narrow spread and favourable median values at these stages demonstrated reliable predictions. In contrast, model performance declined sharply during the rooting stage, with substantial increases in RMSE and PBIAS variability, and negative NSE values in validation (XGB: − 1.846, RF: − 3.535), suggesting unstable predictions. This result can be attributed to the very low and irregular CH₄ emissions during the rooting stage, where even small absolute errors were disproportionately reflected in the performance metrics. This is also related to the fact that a sufficiently reduced environment has not yet been established immediately after transplantation. Before methanogens become fully active, alternative electron acceptors such as oxygen, nitrate, manganese (Mn), and iron (Fe) have not yet been completely depleted, likely resulting in minimal methane production^43^. The distributions shown in Fig. 2 are based on performance metrics calculated from the validation datasets across ten repeated 8:2 random splits.

**Fig. 3:**
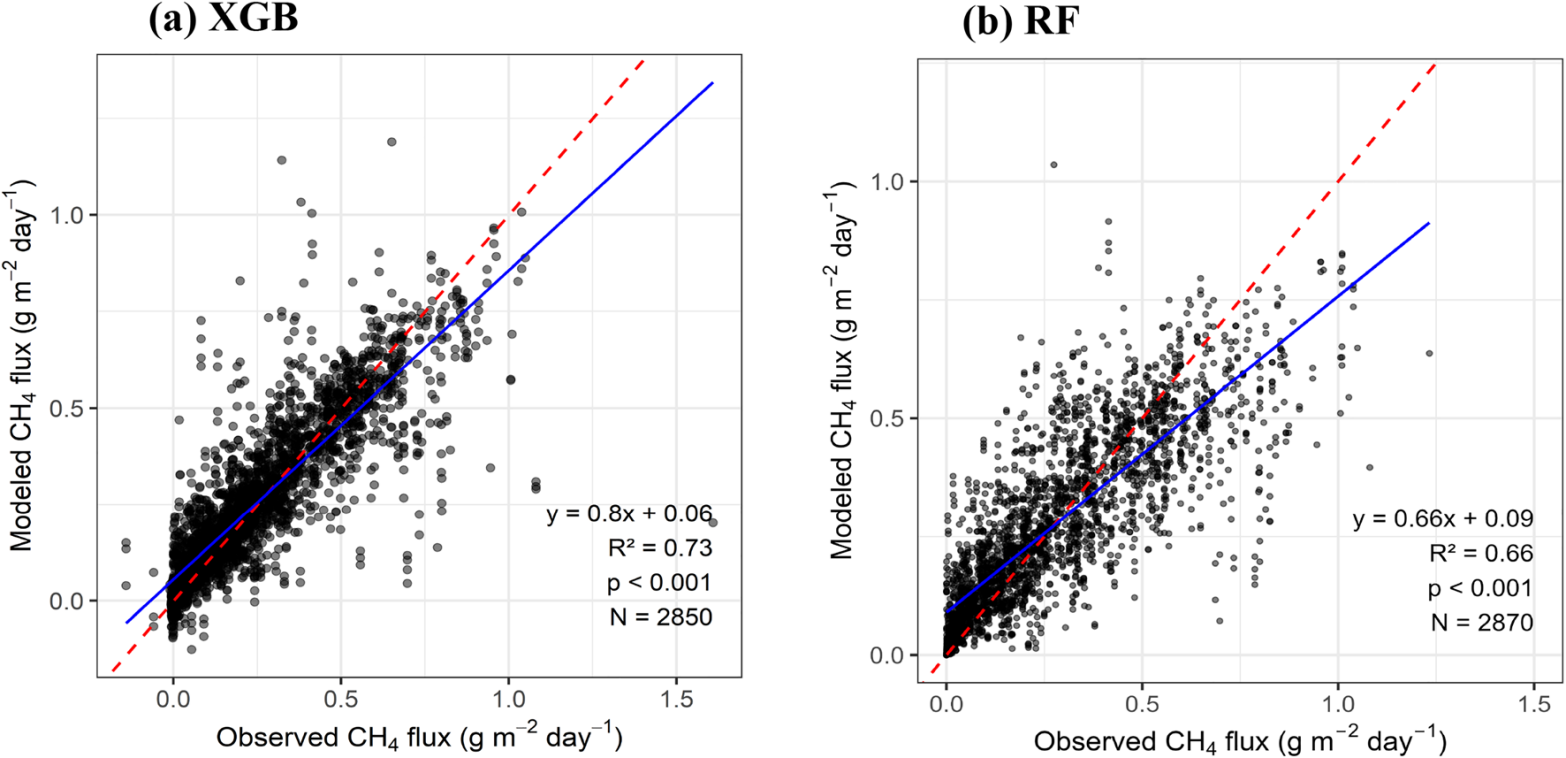
Validation of modeled seasonal total CH₄ fluxes against observations using (**a**) extreme gradient boosting (XGB) and (**b**) random forest (RF). Regression equation, coefficient of determination (R2), significance level (p-value), and sample size (N) are shown.

To compare the model predictions with actual observations, scatter plots were constructed and regression analyses were performed (see in Fig. 3**.**). Both models exhibited clear linear correlations with the observed methane emissions (XGB, R^2^ = 0.73; RF, R^2^ = 0.66; P < 0.001), demonstrating their ability to reproduce the variability in methane fluxes driven by multiple interacting factors. More specifically, the regression slope of the XGB model was 0.80, closer to 1, with relatively narrow data dispersion, indicating stronger predictive accuracy, even in high emission ranges. In contrast, the RF model showed a lower regression slope (0.66) and broader scatter, capturing the overall trend reliably but with reduced accuracy for extreme values. This reflects the strength of the boosting-based XGB algorithm in learning nonlinear patterns, including outliers, whereas the bagging-based RF is more advantageous in reducing variance and achieving stability. Some outliers observed in both models are attributable to the inherent measurement uncertainties of the closed-chamber method, where errors may arise from sampling procedures, timing, and chamber placement^44^. Moreover, given the strong spatiotemporal variability in methane emissions^5^, even subtle environmental differences under similar conditions are likely to contribute to the observed dispersion. Notably, the slight decline in model performance during the rooting stage may be related to growth-stage-specific emission patterns, as well as biases introduced during data partitioning that disproportionately emphasize certain conditions. These outliers can be interpreted as the result of the combined effects of the measurement limitations, structural data biases, and stage-dependent emission characteristics. This highlights the importance of data preprocessing and quality control prior to model training, as reducing input uncertainties is critical for enhancing the model performance.

### Regional simulation of CH₄ emissions in Gimje

Table 4 summarizes the cumulative methane emission estimates, emission factors, and associated uncertainties derived from the XGB and RF models for all the rice paddies in the Gimje region. The XGB model estimated an annual average emission factor of 2.64 kg CH₄ ha⁻^1^ d⁻^1^ and a total annual emission of 7.51 Gg CH₄ yr⁻^1^, whereas the RF model yielded slightly lower values of 2.35 kg CH₄ ha⁻^1^ d⁻^1^ and 6.68 Gg CH₄ yr⁻^1^, respectively. Based on the 95% confidence interval, XGB ranged from 6.54–8.49 Gg, while RF ranged from 5.89–7.47 Gg. Although the estimates of the two models partially overlapped, XGB consistently produced higher values overall. The relative uncertainties were ± 13% for XGB and ± 11.9% for RF, which fell within a statistically stable range compared with the default uncertainty of ± 50% suggested by the IPCC guidelines^45^. The slightly lower uncertainty of RF reflects its tendency to emphasize mean trends, suggesting its potential advantage for broader model generalization. Conversely, XGB achieved higher predictive performance while maintaining an acceptable uncertainty level of ± 13%, indicating its applicability for regional-scale methane emission estimation.

**Table 4:** Predicted CH₄ emission factors and total emissions in Gimje region by XGB and RF models with 95% confidence intervals.

Variables	Models	Predicted mean	Lower 95%	Upper 95%	Range
Emission factor (kg ha⁻^1^ d⁻^1^)	XGB	2.64	2.30	2.98	0.68
	RF	2.35	2.07	2.62	0.55
Total emission (Gg CH₄ yr⁻^1^)	XGB	7.51	6.54	8.49	1.95
	RF	6.68	5.89	7.47	1.58
Emission factor (kg ha⁻^1^ d⁻^1^)	XGB	7.51	6.54	8.49	1.95
	RF	6.68	5.89	7.47	1.58

The official paddy area is 20,815 ha^25^, while the regional totals are aggregated over the mapped paddy mask of 20,769 ha; Relative uncertainties: ± 13% for XGB and ± 11.9% for RF).

**Fig. 4:**
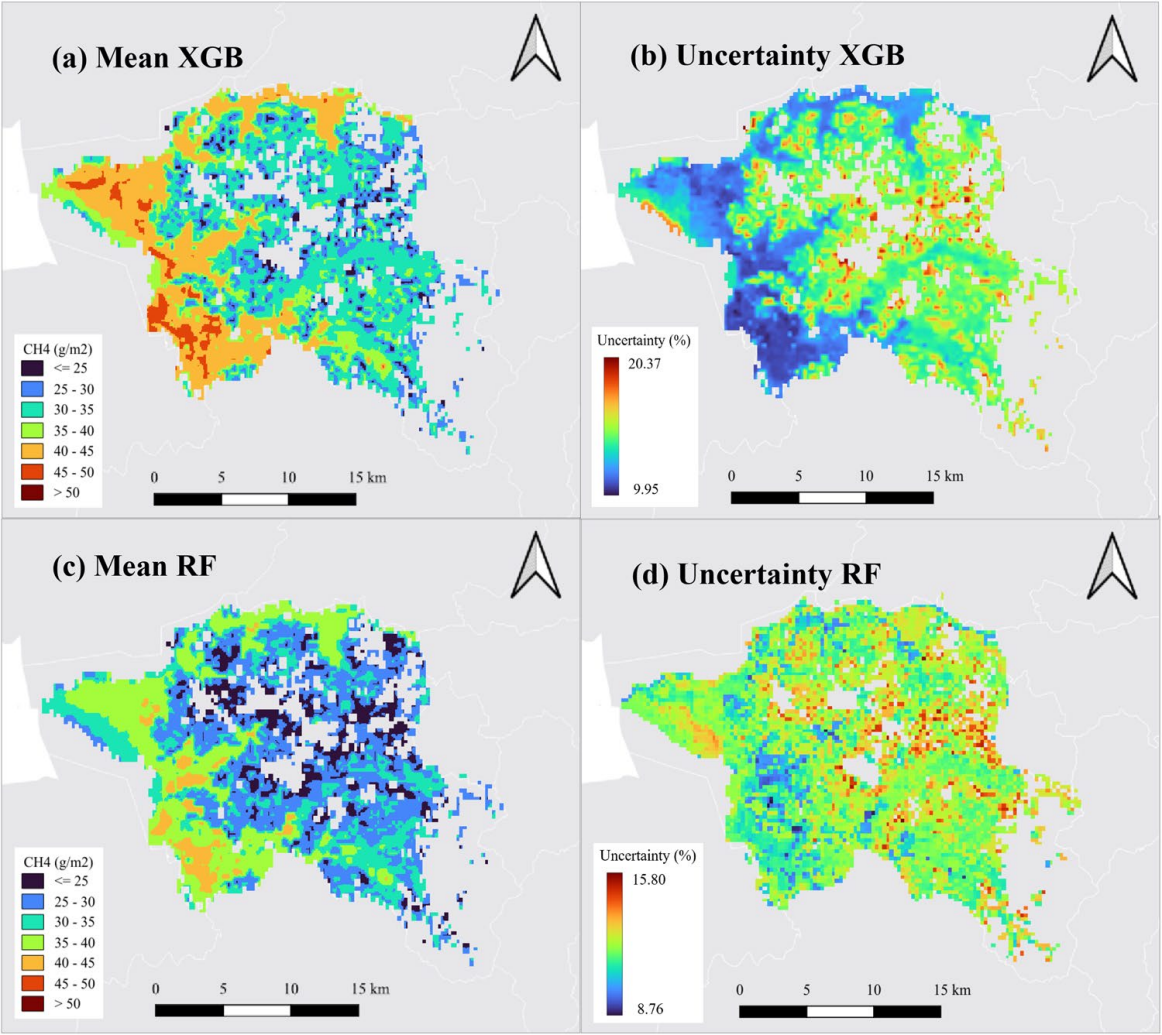
Spatial distribution of cumulative CH₄ emissions in Gimje, Korea, predicted by the XGB model (**a**–**b**) and the RF model (**c**–**d**), showing the mean and relative uncertainty (± %) based on the 95% confidence interval.

These numerical results are consistent with the spatial distribution patterns (shown in Fig. 4). When the trained models were applied to rice paddies across Gimje, the annual cumulative CH₄ emissions ranged from 0 to 61 g m2, revealing pronounced spatial heterogeneity. In the XGB model, the upper-bound estimates indicated concentrated high emissions in the western region and relatively low emissions in the eastern region. Notably, emission hotspots exceeding 50 g m2 were predominantly localized in the west, with persisting strong spatial contrasts, even in the mean estimates. Under lower-bound estimates, emissions were generally reduced to below 20 g m2 across most areas. However, certain western sites still display comparatively elevated levels. This demonstrates that the XGB captured high-emission zones sensitively while maintaining consistent spatial patterns across both upper and lower ranges. In contrast, the RF model produced upper-bound estimates that were comparable to the mean predictions of the XGB, whereas the lower-bound estimates showed a substantial reduction, with most areas falling below 20 g m2.

**Fig. 5:**
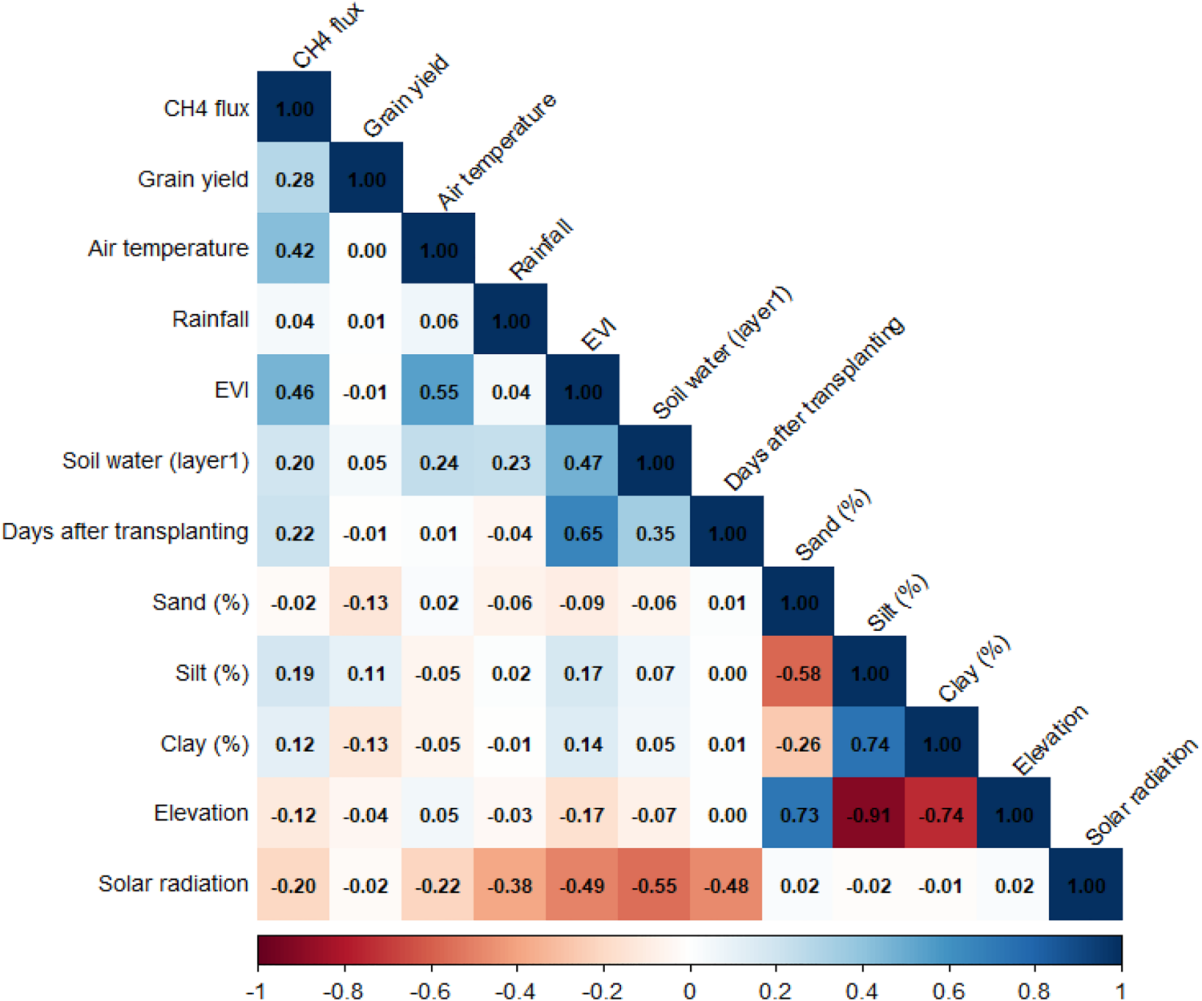
Matrix of correlations between CH₄ flux and explanatory variables.

To analyze regional emission trends, we conducted correlation analyses between the key variables of the model and observed methane emissions (Fig. 5). In Gimje, CH₄ flux showed significant correlations with crop productivity, as well as meteorological, soil, and growth-related factors. Notably, grain yield (gy, r = 0.28***) exhibited the strongest association with methane emissions. This suggests that higher rice productivity increases biomass and photosynthetic products, thereby enhancing root exudation of organic substrates that serve as precursors for methanogenesis. Such mechanisms have been consistently reported in previous studies^46^ (Yagi and Minami 1990b), aligning with the finding that baseline methane emissions are higher in highly productive rice-growing regions. Mean air temperature (tavg, r = 0.42***) was also positively correlated with methane emissions. Elevated temperatures are known to stimulate anaerobic microbial metabolism and methanogen activity in soils^6,47^, while simultaneously promoting rice growth, thereby amplifying methane production. Soil moisture (volumetric soil water, r = 0.20***) reflected reductive soil conditions and provided a favorable environment for methane generation^48^. In addition, days after transplanting (DAT, r = 0.22***) captured the progressive increase in methane emissions associated with crop development. Similarly, the Enhanced Vegetation Index (EVI, r = 0.46***) represented photosynthetic potential and biomass accumulation, supporting the physiological process by which vigorous growth enhances methane emissions^34^. Conversely, surface net solar radiation (r = –0.20***) and elevation (r = –0.12***) were negatively correlated with methane flux. Although higher radiation is generally associated with enhanced methane production through increased root-derived substrates ^6^, in the study area, this mechanism appeared to be offset by water management practices (MD and AWD), this pattern was not observed in the study area. Elevation likely reflects more favourable drainage, leading to reduced methanogenesis. With respect to soil texture, clay (r = 0.12***) and silt (r = 0.19***) content were significantly and positively correlated with methane emissions, indicating that fine-textured soils are more conducive to methane generation. In contrast, sand showed no significant relationship. This may be explained by the limited variability in sand content within the experimental area, which hinders the detection of distinct effects. Overall, the spatial variability of methane emissions observed in Gimje can be attributed not only to climatic factors but also to the combined influence of productivity, soil moisture, soil physical properties, and management practices. This interpretation was consistent with the spatial patterns simulated by the regional model (see in Fig. 5). The correlations between methane emissions and environmental variables in Gimje were broadly consistent with the mechanistic findings of previous studies, supporting their generalizability beyond local conditions. Nonetheless, because bivariate correlation analyses cannot fully capture nonlinear relationships or establish causality, further validation is required to determine whether these mechanisms hold consistently across other regions when scaling the model up to the national level.

**Fig. 6:**
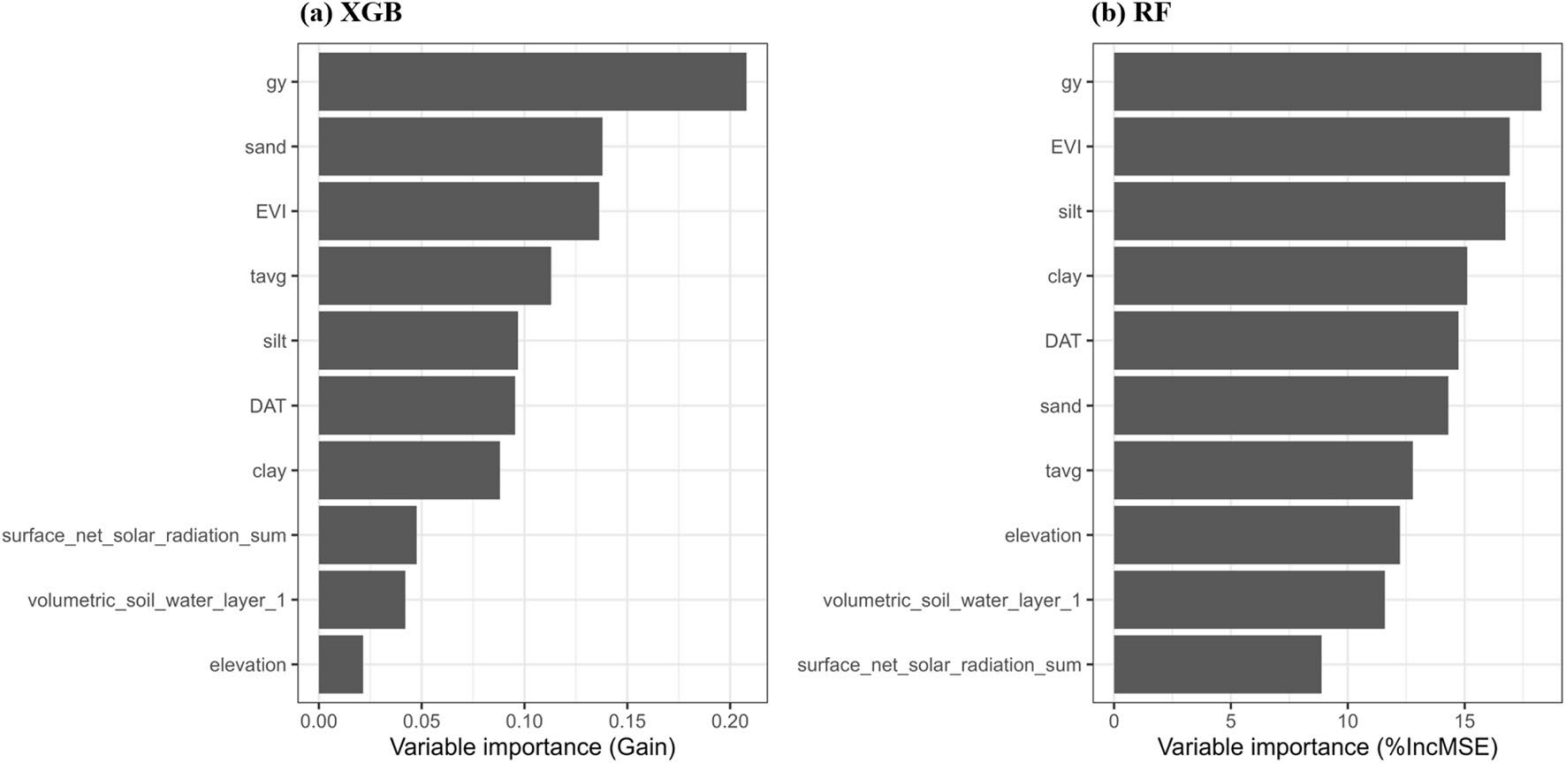
Variable importance derived from the XGB and RF models. Importance for (**a**) the XGB model is quantified by the Gain metric, and (**b**) for the RF model by the percentage increase in mean squared error (%IncMSE). Values represent the average importance calculated across 10 repeated 8:2 train–validation splits.

To find the relative contribution of explanatory variables beyond bivariate correlations (Fig. 5), we conducted model-derived variable importance analysis using XGB (Gain) and RF (%IncMSE) (Fig. 6). In the XGB model, grain yield (gy) showed the highest importance (Gain = 0.214), followed by EVI (0.187) and mean air temperature (tavg, 0.163). Days after transplanting (DAT, 0.141) and volumetric soil water (0.098) also contributed substantially, whereas soil texture variables (silt: 0.072; clay: 0.061; sand: 0.044) and elevation (0.020) showed relatively lower importance. In the RF model, gy showed the highest importance (%IncMSE = 18.6%), followed by EVI (16.9%), tavg (15.4%), and DAT (13.8%). Soil moisture and texture variables exhibited moderate contributions (silt: 8.7%; clay: 7.9%; sand: 6.2%), while elevation and solar radiation showed comparatively smaller effects. These patterns are consistent with the correlations shown in Fig. 5, where rice yield and growth-related variables (gy, EVI, DAT) were positively associated with CH_4_ flux. Temperature also remained influential in both models, supporting its role in regulating methane production. Compared with correlation analysis, the importance metrics provide indication of how much each variable contributes within the predictive model.

### Comparison with national inventory (Tier 2) and implications

In Gimje, the area-based CH₄ emission factors were estimated at 2.64 kg CH₄ ha⁻1 d⁻1 with the XGB model and 2.35 kg CH₄ ha⁻1 d⁻1 with the RF model (Table 4), values that are comparable to or slightly higher than the Tier 2 country-specific factor of 2.32 kg CH₄ ha⁻1 d⁻1 (95% CI: 1.82–2.82) used in the national greenhouse gas inventory^49,50^. Although both model estimates fall within the confidence interval of the Tier 2 factor, Gimje’s characteristics—lowland clay soils, high yields, and intensive cultivation—explain the regionally elevated emissions relative to the national average. Thus, the emission factors derived in this study represent realistic values that reflect regional conditions rather than generalized national averages. Differences also emerged between the two machine learning models. XGB estimated a total annual emission of 7.51 Gg yr⁻1, higher than RF’s estimate of 6.68 Gg yr⁻1, reflecting the boosting framework’s sensitivity to nonlinear interactions and extreme input values. In contrast, RF provided more conservative estimates, with lower uncertainty (± 11.9%) than XGB (± 13%), which is consistent with its bagging-based stability in reproducing average emission levels across growth stages. These differences highlight how the models captured spatial heterogeneity in Gimje according to their algorithmic characteristics. These findings suggest that applying a single Tier 2 factor nationwide cannot adequately capture regional heterogeneity in CH₄ emissions. In large-scale, high-intensity rice cultivation areas, such as Gimje, emissions are likely to be underestimated when using uniform factors. Collectively, these results indicate that spatially explicit modelling approaches using RF and XGB can enhance the reliability of national CH₄ emission estimates and contribute to region-specific mitigation strategies.

### Limitations of this study

Machine learning techniques were applied to three years of observed CH₄ flux data from Gimje to estimate regional methane emissions by incorporating soil, weather, crop growth, and management factors. This approach offers greater precision and realism than the conventional Tier 2 emission factor method. Nevertheless, several limitations remain. First, the training dataset was derived from point-scale observations within Gimje, representing approximately 2% of Korea’s total rice-growing area. Although the comparison demonstrated reasonable consistency between the regional modelling results and the national Tier 2 factors at this local scale, the findings cannot be considered nationally representative. National spatial coverage is required to reduce regional uncertainty. Second, this study compared regionally modelled emission factors with Korea’s country-specific Tier 2 factors. Korea’s national greenhouse gas inventory currently relies on country-specific Tier 2 emission factors derived from short-term experiments in limited regions and cultivars, uniformly applied across all rice paddies. While this ensures simplicity and comparability, it does not account for regional variability caused by soil, climate, water management, and varietal differences. Although this study addressed part of this gap through the Gimje dataset, the results are not fully representative at the national scale. The three-year observation period was insufficient to capture interannual climate variability and long-term trends, and data from diverse soils, climates, and cultivation systems were excluded. Key management practices such as mid-season drainage, straw incorporation, and fertilization were also underrepresented due to limited data availability, potentially leading to biased estimates. Finally, although this study demonstrates the feasibility of machine learning as a step toward Tier 3-level estimation, further progress requires broader data integration and model intercomparison. Establishing a comprehensive database from published studies and long-term multi-regional observations is essential to reduce uncertainty and advance the national inventory toward Tier 3 methodologies.

## Conclusion

This study estimated regional methane (CH₄) emissions from paddy fields in Gimje, Korea using machine learning models, XGBoost (XGB) and Random Forest (RF), trained with three years of observations. Both models achieved Nash–Sutcliffe efficiency values above 0.5. XGB better captured extreme values and temporal variability, while RF more reliably reproduced mean trends, showing complementary strengths. Applied to the Gimje paddy area, mean emission factors were 2.64 and 2.35 kg CH₄ ha⁻1 d⁻1 for XGB and RF, respectively, comparable to or slightly higher than the Tier 2 country-specific factor (2.32). XGB identified high-emission hotspots with greater sensitivity, whereas RF produced smoother patterns with lower uncertainty. Overall, these results demonstrate the feasibility of machine learning for regional CH₄ estimation and its potential as a Tier 3 method to complement Tier 2 inventories. Future work should combine multi-year, multi-site data to enable nationwide modeling and a more robust inventory framework.

## Supplementary Information

Supplementary Information.

## Data Availability

The research data supporting the findings of this study are openly available in the Zenodo repository at [10.5281/zenodo.17111992] (10.5281/zenodo.17111992).
