# Supplementary material for: Regional machine learning-based estimation of methane emissions from rice cultivation in South Korea
Source: Sci Rep. 2026 Apr 27;16:19487. doi: 10.1038/s41598-026-49883-4 (PMC13287486; doi:10.1038/s41598-026-49883-4)
Supplement: Supplementary file 1 — Supplementary Information. [file 41598_2026_49883_MOESM1_ESM.docx]

**Supplemental information**

Regional Machine Learning-Based Estimation of Methane Emissions from Rice Cultivation in South Korea

**Hyoung Seok Lee, Jong Mun Lee, So Ra Lee, Hye Ran Park, Min Ji Lee and Young-Jae Jeong**

Table S1. Water management scenarios.

| No. | Water management | Description | Code |
| --- | --- | --- | --- |
| 1 | Continuously flooding | 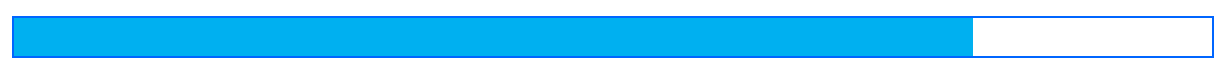 | CF |
| 2 | Mid-drainage | 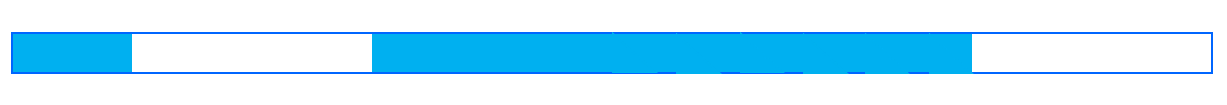 | MD |
| 3 | Alternative wetting and drying | 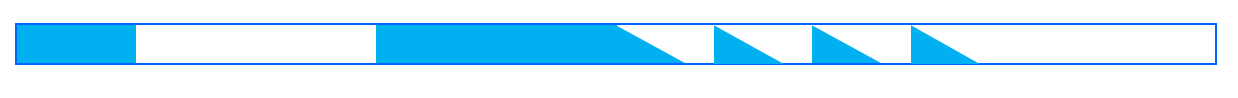 | MD+  AWD |

S1. Water management methods (CF, MD, MD+AWD).

Continuously flooding (CF) refers to the practice of keeping paddy fields submerged with water throughout the entire growing season, which ensures stable water supply but is associated with high methane emissions. Mid-drainage (MD) involves draining water for a certain period approximately 30 days after transplanting, allowing soil aeration and thereby reducing methane release. MD combined with alternate wetting and drying (MD+AWD) is a multi-aeration practice in which, following MD, intermittent wetting and drying is applied after the heading stage to further enhance water-use efficiency and mitigate greenhouse gas emissions.


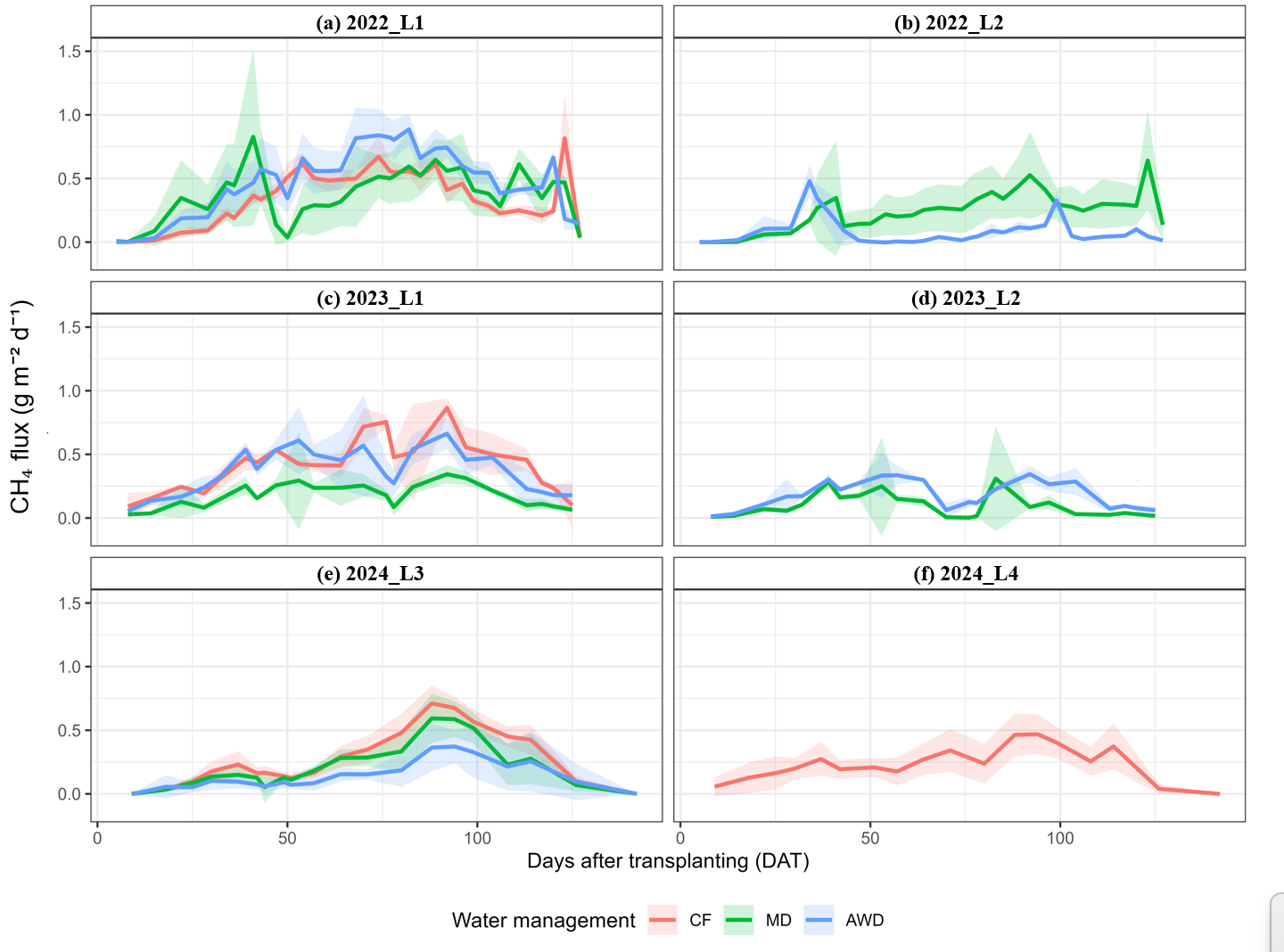


Fig. S1. Time series of CH₄ flux (mean ± SD) under continuous flooding (CF), mid-drainage (MD), and MD+AWD across six year-location combinations (2022–2024).

Table S2. Summary statistics of observed CH₄ flux (mean ± SD) under different water management regimes at each site–year combination.

| Year-Location | Replicates | Number of Sampling Events | Water management | CH_4_ flux (g m^-2^ d^-1^) |
| --- | --- | --- | --- | --- |
| 2022_L1 | 3 | 32 | CF | 0.36 ± 0.225 |
| 2022_L1 | 3 | 32 | MD | 0.38 ± 0.279 |
| 2022_L1 | 3 | 32 | AWD | 0.48 ± 0.276 |
| 2022_L2 | 6 | 32 | MD | 0.24 ± 0.227 |
| 2022_L2 | 3 | 32 | AWD | 0.08 ± 0.118 |
| 2023_L1 | 3 | 22 | CF | 0.41 ± 0.225 |
| 2023_L1 | 3 | 22 | MD | 0.17 ± 0.124 |
| 2023_L1 | 3 | 22 | AWD | 0.36 ± 0.209 |
| 2023_L2 | 3 | 22 | MD | 0.09 ± 0.136 |
| 2023_L2 | 3 | 22 | AWD | 0.18 ± 0.120 |
| 2024_L3 | 3 | 20 | CF | 0.26 ± 0.223 |
| 2024_L3 | 6 | 20 | MD | 0.20 ± 0.200 |
| 2024_L3 | 6 | 20 | AWD | 0.14 ± 0.151 |
| 2024_L4 | 12 | 18 | CF | 0.23 ± 0.177 |

S2. Detailed Gas Sampling Protocol and Schedule.

Gas sampling for methane (CH_4_) flux was conducted throughout the rice cultivation period, spanning approximately 137 days. To ensure high temporal resolution and capture the dynamic nature of emissions, sampling was performed at least once a week, with a total of 18 to 32 sampling events recorded across the different study sites (Table S2). All gas collection was strictly scheduled between 10:00 and 12:00 h (Fig. S2), a specific time window chosen because the CH₄ emission rate during these hours has been shown to closely represent the daily average emission rate under field conditions (Jeong et al., 2022; Jeong et al., 2018). The sampling followed a standardized static chamber method. Upon placing the chamber, an initial air sample was collected from the headspace using a 60 mL syringe, and the initial internal temperature was recorded. The chamber was then sealed for exactly 30 minutes. After this closure period, a second air sample was collected using another 60 mL syringe, accompanied by a final temperature measurement. To ensure the accuracy of the flux calculation, the effective headspace volume was determined by measuring the height from the water surface to the chamber lid at each event. Additionally, environmental parameters such as soil temperature, water temperature, and oxidation-reduction potential (Eh) were monitored concurrently to evaluate the factors influencing flux variations.


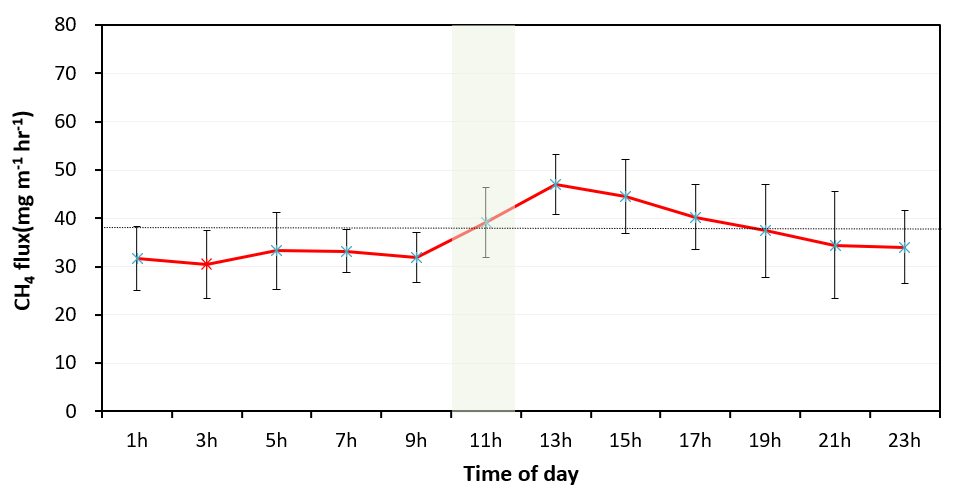


Fig. S2. Diurnal variation of methane (CH₄) flux (Jeong et al. 2018).
